# Improving the Antimicrobial and Mechanical Properties of Epoxy Resins via Nanomodification: An Overview

**DOI:** 10.3390/molecules26175426

**Published:** 2021-09-06

**Authors:** Roberta Bertani, Alessandra Bartolozzi, Alessandro Pontefisso, Marino Quaresimin, Michele Zappalorto

**Affiliations:** 1 Department of Industrial Engineering, University of Padova, Via F. Marzolo 9, 35131 Padova, Italy; alessandra.bartolozzi@unipd.it; 2 Department of Management and Engineering, University of Padova, stradella S. Nicola 3, 36100 Vicenza, Italy; alessandro.pontefisso@unipd.it (A.P.); marino.quaresimin@unipd.it (M.Q.)

**Keywords:** epoxy resins, nanocomposites, antimicrobial activity, mechanical properties, organic and inorganic nanofillers

## Abstract

The main purpose of this work is to provide a comprehensive overview on the preparation of multifunctional epoxies, with improved antimicrobial activity and enhanced mechanical properties through nanomodification. In the first section, we focus on the approaches to achieve antimicrobial activity, as well as on the methods used to evaluate their efficacy against bacteria and fungi. Relevant application examples are also discussed, with particular reference to antifouling and anticorrosion coatings for marine environments, dental applications, antimicrobial fibers and fabrics, and others. Subsequently, we discuss the mechanical behaviors of nanomodified epoxies with improved antimicrobial properties, analyzing the typical damage mechanisms leading to the significant toughening effect of nanomodification. Some examples of mechanical properties of nanomodified polymers are provided. Eventually, the possibility of achieving, at the same time, antimicrobial and mechanical improvement capabilities by nanomodification with nanoclay is discussed, with reference to both nanomodified epoxies and glass/epoxy composite laminates. According to the literature, a nanomodified epoxy can successfully exhibit antibacterial properties, while increasing its fracture toughness, even though its tensile strength may decrease. As for laminates—obtaining antibacterial properties is not followed by improved interlaminar properties.

## 1. Introduction

There is a large amount of literature on the preparation of multifunctional nanocomposites with improved mechanical and antimicrobial properties. In this work, we focus on epoxy resins, due to their wide applications in different fields and relatively easy chemical modification procedures aimed at developing innovative solutions for surface modification, coating technology, and bulk materials.

The emergence of novel pathogenic viruses and antibiotic resistant human pathogens (bacteria, fungi, protozoa, parasites), due to their highly-mutative capacities and rapid morphological changes, have prompted research into alternative antimicrobial materials [1,2,3], including plastics with long-term biocidal activity in nanostructured “bulk” material, and bioactive surfaces, whose efficacy involves a direct contact (and subsequent localized reaction) for microbial inactivation.

Polymer nanocomposites are a class of materials characterized by outstanding mechanical and functional properties, achieved with limited filler content, compared with composites with microsized reinforcements. The term “nanocomposite” refers to the size of the reinforcement, which has at least one dimension on the nanometer scale, leading to an extremely high surface-to-volume ratio that gives rise to the so-called “nano-effect” [4].

Nanomaterials benefit from having dimensions similar or lesser than the target bacteria (or viruses), allowing, in principle, for intimate interactions. However, to maintain this characteristic, it is necessary to prevent the formation of nanofiller agglomerates: it can be achieved through good dispersion in a matrix and/or if the nanoparticles have electric charges, at a neutral pH, that repel them reciprocally and, if possible, attract the microorganisms [5].

One consequence of chemical functionalization of nanoreinforcements, is the formation of an interphase: a material phase at the matrix-reinforcement interface whose properties differ from those of its constituents [6]. This phase is proportional to the contact surface between the filler and matrix; therefore, it reaches remarkable volume content due to the nano-effect, and effectively affects the overall composite properties [7].

The antimicrobial agent can be grafted to the polymer surface (through suitable chemical synthons as linkers), physically adsorbed (through the formation of a large number of non-covalent or electrostatic interactions, giving rise to self-assembled structures), through a surface reaction starting from a precursor forming an antimicrobial film [8,9], or by incorporating antimicrobial agents directly into the epoxy resin, which could be formed in the presence of it [10]. In any case, the materials with antimicrobial activity need to be tailored according to the specific applications.

Given the importance of the above-mentioned topics for many industrial fields of application, the main purpose of this work is to comprehensively discuss the possible advantages deriving from the nanomodification of epoxy polymers, in terms of both antimicrobial activities and mechanical properties.

## 2. Enhancement of Antimicrobial Properties via Nanomodification of Epoxies

### 2.1. Antimicrobial Agents and the Effects of Immobilization on Their Activities

In this section, examples of the most studied approaches to achieve epoxy nanocomposites exhibiting antimicrobial activity are presented, including also the methods used to evaluate their efficacy against bacteria and fungi, which, even if based on standard parameters, must often be optimized with respect to the specific material and microorganism. In particular, a distinction is proposed between nanocomposites, containing (in the epoxy polymeric matrix) a dispersion of nanofillers of different chemical natures (such as metal nanoparticles or clays), and nanohybrids, where covalent bonds between the epoxy polymeric matrix and organic functional moieties are present.

**Scheme 1:**
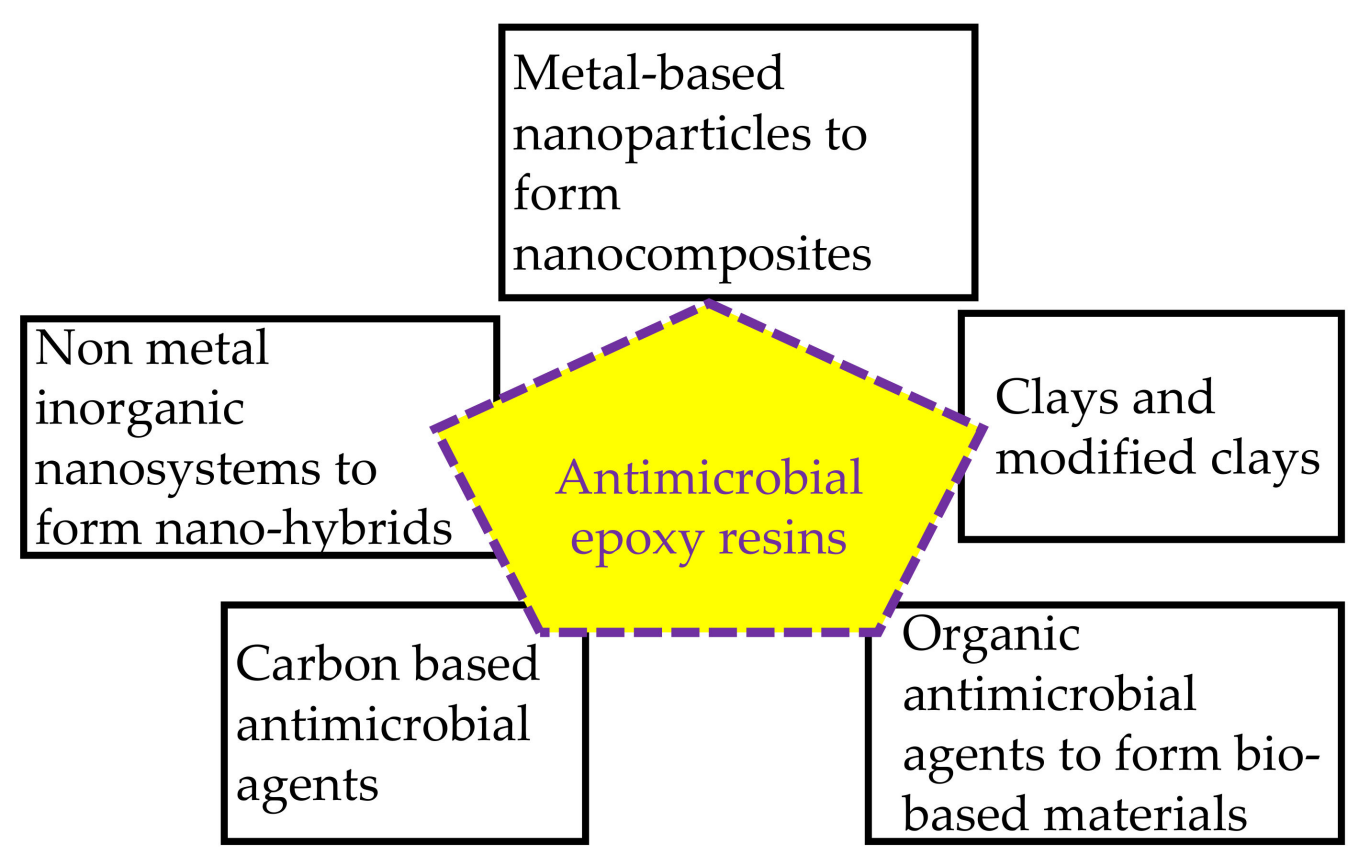
Different approaches to impart antimicrobial properties to epoxy resins.

A wide variety of antimicrobial agents have been immobilized in epoxy resins, typically as nanoparticles (NPs) containing metal systems (such as silver, copper, ZnO, or TiO_2_), organic compounds (such as quaternary ammonium compounds, guanidinium-based molecules, or chitosan), inorganic nanoparticles, carbon-based systems, and clays, depending on the specific application and set of properties required [11] (Scheme 1).

It is valuable to consider the antimicrobial mode of action, i.e., how it can be modified by immobilization, depending on the type, dimension, and physiology of the bacteria of interest [9].

Three mechanisms of antimicrobial activity could be distinguished based on: (i) surfaces that release antimicrobials; (ii) surfaces able to kill adhering bacteria directly upon adhesion without release of antimicrobials; (iii) surfaces that are non-adhesive for bacteria. Even if the exact antibacterial activity of a specific agent against a specific microorganism has not been completely elucidated, some common processes were considered: (i) physical lysing of the membranes; (ii) charge disruption of the membrane potential; (iii) solubilization of the membrane phospholipids yielding holes; (iv) modification of transport properties of the membrane, inducing changes in the metabolic redox processes. For example, microorganisms have an enzymatic antioxidant defense system to control reactive oxygen species (ROS) formation (i.e., superoxide and hydroxyl radicals, hydrogen peroxide, single oxygen) in an equilibrium that can be altered by the presence of metal NPs, which can spread through the membrane and affect enzymatic activities and cellular structures [12].

Ag, ZnO, and TiO_2_ NPs can interact with the cell by electrostatic attraction, van der Waals forces, and hydrophobic interaction. Different microorganisms have specific cell wall compositions and properties: gram-positive bacteria (such as *Staphylococcus aureus* or *Enterococcus faecalis*) contain many layers of peptidoglycan and teichoic acid (medium thickness 20–50 nm); gram-negative bacteria (such as *Escherichia coli*) have thin peptidoglycan layers and outer membranes containing lipopolysaccharides and lipoproteins; fungi and yeast cell walls mainly contain chitin and polysaccharides; viruses have protein shells (capsids) [13]. Thus, the membrane damage by antimicrobial agents must occur through different mechanisms. It was observed that metal NPs (usually with positive charges) are more active against Gram-positive bacteria, having higher permeability and negative charges on the surface, that attract NPs, in comparison with Gram-negative bacteria, where the double membrane, with a lower negative charge, acts as a physical barrier against hydrophobic compounds [14].

Surface adhesion is one of the key steps of colonization and biofilm formation; the latter is synergistic by maintaining cell-to-cell proximity and communication via quorum sensing [15], thus, synchronizing gene expression. To inhibit quorum sensing in vivo, the inhibitory agent must be, in general, of low molecular mass, and specific for the regulators involved. Examples are Ag NPs, garlic extract, and small molecules acting as enzyme inhibitors (i.e., chitosan) [16].

In general, we could attempt to understand if the antimicrobial activity originates from the leaching of active agents, from the surface itself or from a mass transport of bacteria on the surface. On the basis of these considerations, the most appropriate test methods to evaluate antimicrobial activity can be selected, and/or compared. In any case the specific conditions of the test deserve attention and critical evaluation [17] (Table 1).

The most common test approaches are reported in the following, and are often optimized for specific systems [18]:

1. Observation of a zone of inhibition: the procedure involves placing a portion of the epoxy resin loading of the antimicrobial agent in contact with a growth media loaded with bacteria. As the antimicrobial agent elutes from the resin into the media, a region with an inhibited growth of bacteria is observed. The data must be compared with the same experiment carried out with the same epoxy resin, which does not contain the antimicrobial agent [9,19]. The method is not able to distinguish between bacteriostatic (delay of the bacterial growth) and bactericidal (inhibition of bacterial growth) effects, both depending on several factors; usually, the antimicrobial effectiveness involves both activities.
2. Immersive method: the procedure involves the immersion of the bioactive sample into the media containing bacteria, under stirring, and the measure of the colony-forming units (CFUs) derived from the inoculum solution. The same experiment is carried out with a control (comparing the measured CFU), even if its surface could be significantly different from that of the sample, which could be positively charged if an ammonium moiety is the antimicrobial agent, thus strongly influencing the cell-surface adhesion. The method was developed as a standard procedure by ASTM (ASTM E2149).
3. Direct inoculation: the Japanese Industry Standard (JIS Z-2801) involves placing a droplet of inoculum directly on the surface of the sample and, after a suitable inoculation time, the release of cells, under defined conditions, and enumeration as CFU.
4. Surface growth method: it involves the application of a thin film of pathogens on the surface of the sample, following its growth, compared to that of the control, for a long time, typically days, thus evaluating the surface ability to form a biofilm.

The above-mentioned methods could have limitations when the bacteria are too low to be enumerated. This can be due to microorganisms that are viable, but not culturable under the used conditions, due to stresses like starvation or low temperatures. It was suggested that this is a strategy adopted by bacteria to survive under unfavorable environmental conditions. In this situation, the right test conditions must be found, adapting the composition of the medium, times, etc., for the specific species/strain/phenotype, etc., for their resuscitation and enumeration [20].

Although growth enumeration is the most familiar method for achieving quantitative data, other methods were applied to detect the viability of microbes, including optical density (OD) and luminescence, by using fluorescent stains with confocal or epi-fluorescent microscopy, or in combination with flow cytometry. This approach takes advantage of the bioluminescence generated within the metabolic activity of cells, thus, achieving information concerning the mechanisms of action [21].

### 2.2. Nanoscale Antimicrobial Agents

#### 2.2.1. Metal-Based and Inorganic Nano-Modifiers

As for inorganic nanoparticles, two types of materials can be considered:

(i). Nanocomposites, prepared by dispersion of nanoparticles into the epoxy mixture, where the interactions are essentially of physical/electrostatic nature [22]; and
(ii). Nanohybrid materials, where the inorganic phase is formed in situ, achieving covalent chemical bonds between the epoxy matrix and the inorganic part [23] through sol–gel processes or reaction of functionalized nanoparticles with the epoxy mixture.

(a) Nanocomposites. Since ancient times, materials containing silver or copper have been used as antimicrobial agents; nowadays metallic (i.e., Ag and Cu) and metal-oxide (i.e., ZnO, CuO, NiO, TiO_2_) nanoparticles provide strong antimicrobial activity in small dosages against a wide variety of microorganisms due to their dimensions, shapes, charges, high ratio of surface areas to mass, and high reactivities [14,24,25,26,27,28,29,30,31]. Due to electronic, optical, and magnetic properties, multi-metallic nanoparticles were recently proposed as innovative antimicrobial agents, which could help to overcome antimicrobial resistance [32]. Chitosan and Chitosan/Ag NPs were also proposed as promising alternatives to overcome antimicrobial resistance in medical devices [33].

The properties of nanoparticles, in terms of morphology and size, strongly depend on the synthesis methods used [14], which could be physical (i.e., evaporation/condensation for Ag, magnetron sputtering, or mechanochemical processes for ZnO, microwave-thermal methods, and photoreduction processes for Ag or CuO, pulsed laser ablation for ZnO), chemical (i.e., atomic layer deposition for TiO_2_, chemical reduction for Ag, sol–gel for TiO_2_, reverse micellar route, or solvothermal or hydrothermal method for ZnO), or biological (through the usage of plant extracts, algae, bacteria, or fungi, which behave as biological nanofactories that can release proteic substances able to chemically reduce metal ions [34,35,36]).

Metal, metal oxide, or silica nanoparticles are usually dispersed into the epoxy, and then the resin is hardened to achieve films or bulk samples [37]. To improve the compatibilization with the epoxy matrix, the metal nanoparticles are often functionalized. Ag NPs prepared by reduction of AgNO_3_ in water with glucose have been treated with chitosan or with poly(acrylonitrile-co-N-vinylformamide) and used as curing agents to prepare epoxy resins [38]. Chitosan was also used to prepare ternary nanocomposites by treating it with TiO_2_ nanoparticles and polyaniline, then added in different amounts to a diluted epoxy resin precursor, subsequently hardened. This allows to obtain films with increased conductivity (with respect TiO_2_ free PANI systems), better thermal and mechanical properties in comparison with the coating of neat epoxy, and showing antibacterial activity against Gram-positive bacteria, more than against Gram-negative ones, reasonably due to their membranes being less susceptible to structural damages. The biocide performance against *Staphylococcus aureus* was explained by the inhibitory effect of TiO_2_ NPs combined with the antimicrobial activity of chitosan [39].

The metal nanoparticle (Ag/CuO) dispersion with strong interaction in epoxy resins was also achieved by using polyethylene glycol 200 for the preparation of samples, which showed increasing antimicrobial activity against *Escherichia coli* with the Ag/CuO nanoparticle amounts. Thermal stability and mechanical properties (with ultimate strength higher than 80%) also significantly increased, with respect to pure epoxy [40].

(b) Nanohybrids. The modification of epoxy resins with silicon moieties (silanes, polysiloxanes, silsesquioxanes, silica, silicates), including sol–gel processes with organoalkoxysilanes as coupling agents, gave hybrid materials with improved and tailored mechanical properties (elastic modulus, hardness, scratch resistance), thermal and flame resistance, and corrosion and antimicrobial protection [23,41]. Nanohybrid coatings were prepared by reacting the epoxy precursor (Aradur) with ZnO core shell nanoparticles functionalized with aminopropyltrimethoxysilane (APTES), bearing a reactive NH_2_ group acting as a coupling agent. The nanocomposites exhibited microbial inhibitory activity against *Pseudomonas aeruginosa, Streptomyces*, and *Aspergillus niger* [42]. A similar strategy was used for TiO_2_ nanoparticles in coatings with high antimicrobial effects against *Staphylococcus aureus* and antifouling properties, depending on the TiO_2_-APTES NP amounts [43]. Ag NPs amino-functionalized with APTES (1, 3, 5 wt%) were used as curing agents for bisphenol A diglycidyl ether (DGEBA). Moreover, reinforced DGEBA coatings functionalized with Ag NPs (1 wt%), was found to be the best formulation for antimicrobial activity (tested against *Pseudomonas aeruginosa*, *Bacillus subtilis*, *Escherichia coli*, *Candida albicans*), mechanical (microhardness from 108 ± 9 for the control, to 176 ± 7) and corrosion resistance [44].

In Table 2, selected nanocomposites based on metal or metal oxide NPs are presented, together with examples where the presence of functional groups on metal NPs allow for stronger interaction between the polymeric matrix and the metal nanoparticles, thus influencing mechanical properties.

#### 2.2.2. Metal Complexes Modifiers

Benzoylhydrazone complexes of Cr(III), Co(II), and Fe(III) have been incorporated into epoxy paints (in 1.0, 1.5 and 2.0 wt%), achieving coatings with good flame retardant characteristics (limiting oxygen index, LOI, ranging from 23 and 30) and very good antimicrobial properties (in particular when containing 1.0 to 2.0 wt% of the Fe(III)-complex) against Gram-positive, Gram-negative, and fungi strains, together with antifouling properties [56].

Epoxy resins bearing into the polymeric chain phenanthroline and Schiff base moieties able to coordinate metal ions (Mn(II), Co(II), Ni(II), Cu(II), Zn(II)) were reported to exhibit very good coating properties (in terms of scratch hardness values and adhesion to the steel substrate) and significant antibacterial activity, which was related to the nature of the metal, with the Cu(II) systems being the most active [57,58,59,60,61].

#### 2.2.3. Nanoclay Modifiers

Clay is a natural material that has been used since ancient times in human and veterinary health formulations. Recently, some exciting research was published concerning the elucidation of the mechanism of cell death induced by some clays (Oregon Blue clays) [62], demonstrating that the release of Fe^2+^, Fe^3+^, Al^3+^, and Ca^2+^ ions damaged the lipopolysaccharides on the outer membrane of *Escherichia coli,* giving protein misfolding and oxidation with activation of a stress-response. H_2_O_2_, formed extracellularly, diffused through the envelope oxidizing intracellular Fe^2+^ to Fe^3+^, giving rise to radicals able to oxidize DNA and proteins, with precipitation of iron oxide and cell death. In this frame, nanoclays can be considered natural fillers bearing metal nano-moieties [10].

Clays, such as montmorillonite, can be easily modified by introducing metal cations (i.e., Cu^2+^ [19] or Ag^+^ [63,64]) or organic cations, such as ammonium or pyridinium salts, changing the charge and hydrophobic/hydrophilic properties. Thus, modified clays, which have been extensively used in the preparation of polymer nanocomposites, gave the final material antimicrobial properties, in bulk [65]. Cu_2_O dots were prepared by reacting Cu(OAc)_2_ with EtOH/NH_3_(aq) and added to octadecyl amine modified montmorillonite, forming nanohybrid particles, which showed excellent antimicrobial activity against both Gram-negative and Gram-positive bacteria, as well as against *Candida albicans* at 3 wt% nanohybrid loading [66]. Moreover, neem oil has been used to modify montmorillonite and prepare epoxy nanocomposites, whose antimicrobial activity was tested by growth curve and zone-of-inhibition analyses against bacteria and fungi (*Bacillus subtilis, Staphylococcus aureus, Klebsiella pneumoniae, Pycnoclavella diminuta, Candida albicans*), observing a neem oil dose-dependent effect [67].

Synthetic zeolite type A containing biocide cations (Ag^+^ and Zn^2+^) was dispersed into epoxy formulation to achieve steel coatings, which were shown to inhibit the growth of *Pseudomonas aeruginosa,* reducing the number of bacteria adhering to the coating; thus, decreasing the corrosive action by bacteria [68].

Recently, halloysite nanotubes were proposed as nano-containers of sodium salicylate or N-(2,4,6-trichlorophenyl)maleimide as antimicrobial agents, to achieve a novel marine antifouling paint [69].

#### 2.2.4. Organic Nanomodifications

Organic nanomodifications of epoxy resins to impart antimicrobial activity were carried out through different strategies:

1. Blending with intrinsically biocidal polymers such as chitosan [70,71] or copolymerization, with organic moieties able to modify the surface polarity, such as PANI [72] or non-isocyanate polyurethane [73], or epoxy polymers made from phenolic branched fatty acids [74,75], or using antibacterial polyether biguanide as a curing agent [76].
2. Incorporating into the epoxy resin of quaternary ammonium moieties, which could be formed by (a) direct reaction of NH groups along the epoxy-resin chain with alkyl bromides [77,78,79], or preparing hybrid siloxane epoxy coatings by reaction of diamine-bearing epoxy moieties with siloxane-bearing amine groups [80]; (b) dispersing quaternary ammonium polyethylenimine nanoparticles previously prepared [81]; (c) preparing polysiloxane quaternary ammonium salts, containing epoxy groups to react with amines [82] and (d) preparing poly(quaternary ammonium salt-epoxy) grafted on Ce or ZnO nanoparticles [83,84].
3. Incorporating biocidal compounds, such as triclosan [85].

#### 2.2.5. Bio-Based Materials

Plant extracts and natural oils are attracting interest as eco-additives of polymer resins, in coating technology, with antimicrobial and antifouling properties [86]. They consist of bioactive antioxidant molecules, based on aromatic hydrocarbons and phenols, bearing O-H and N-H groups, such as terpenes, tannins, and flavonoids. Some examples are presented in Table 3.

Bio-based epoxy polymers, inherently antibacterial, have been prepared by epoxidation of phenolic-branched fatty acids (through preliminary methylation and subsequent reaction with DGEBA) and curing with polyamines. The structure–activity relationship was investigated, observing that the hydrophilicity of the final material (depending on composition) was crucial for the bio-activity, which was not due to small molecules leaching out of the polymer, but originated from higher curing temperatures, forming byproducts, which were beneficial for the antibacterial activity. The material was tested against Gram-positive and -negative strains [75].

Oregano oil was dissolved in DGEBA before curing to achieve an epoxy/oregano oil hybrid material containing micro-sized oil particles with strong interfacial bonding with the epoxy matrix, exhibiting significantly improved impact and fracture properties, decreased tensile strength and modulus, and with antibacterial and antifungal activity [87]. The addition of artemisinin to epoxy resin formulation was reported to impart antimicrobial activity to epoxy resin membranes without damaging mechanical properties [88].

Double bonds in cottonseed oil have been epoxidized and the product was added to DGEBA in different amounts to achieve a bio-based epoxy resin by curing with amine terminated prepolymer. The vegetable oil had a plasticizing effect thus reducing the glass transition temperature; films were transparent and exhibited an inhibition towards bacterial/fungal growth beneath, reasonably due to the in situ formation of ammonium moieties [89].

Fully eco-friendly thermosets from renewable resources have been prepared via reaction of resorcinol diglycidyl ether (resorcinol can be produced from some vegetable resins, such as galbanum and asafoetida, by melting with KOH, and is present in argan oil), with diamine limonene or diamine-allyl-eugenol. It was demonstrated that all novel bio-epoxy resins have comparable mechanical properties to those of benchmark thermosets, with excellent flexural properties in terms of stiffness, and better fracture toughness resistance [90].

#### 2.2.6. Graphene Oxide and Carbon Nanotubes Modifiers

Different formulations of graphene oxide (GO)/epoxy nanocomposites were tested to prepare steel tanks for the storage of biodiesel and GO blends, with improved anticorrosive properties (due to antifouling and antimicrobial properties) being investigated. An LCA study was carried out to optimize the procedure for GO/epoxy nanocomposites preparation in agreement with the Energy Policy Act of 2005 [91]. The influence of oxygen-containing functional groups in epoxy/reduced graphene oxide composite coatings on corrosion and antimicrobial properties was investigated. It was observed that composite coatings with higher amounts of oxygen-containing functional groups showed better antimicrobial activity due to the oxidative stresses induced by oxygen, which could be easily absorbed as a consequence of the high defect densities [92].

With the aim to prepare nanocomposites with efficient antimicrobial activity for advanced applications, from biomedical to marine coatings, biocompatible thermosetting hyperbranched epoxy/Ag-reduced graphene oxide nanocomposites were prepared. It was reported that the new materials inhibited the growth of *Staphylococcus aureus* and *Candida albicans*, with minimum inhibitory concentration of 38 and 41 mg mL^−1^, at 3% wt of the immobilized nanohybrid, and prevented the growth of microalgae *Chlorella* sp. [93].

Again, hyperbranched epoxy/MWCNT-CuO nanohybrids were prepared by sonicating multi-walled carbon nanotubes (MWCNT) with CuO in water. The CNT-CuO hybrid was then dispersed in THF; nystatin (an antifungal medication) was added. The mixture was incorporated in 1, 2, 3 wt% in hyperbranched epoxy resin and was cured with poly(amido amine) to prepare new coatings, which exhibited antimicrobial (against *Staphylococcus aureus*) and antifungal (against *Candida albicans*) activity. In particular, an inhibitory effect within the concentration range 75–54 mg mL^−1^ against *Candida albicans* for the 3 wt% loading material was observed. No growth of microalgae *Chlorella* sp. was observed [94].

### 2.3. Selected Applications

#### 2.3.1. Antifouling and Anticorrosion Coatings

Marine biofouling is an undesirable accumulation of microorganisms, algae, and animals on surfaces in contact with water, which determine a higher frictional resistance for ship hulls due to the roughness and weight increasing, with relevant economic impact. The fouling process involves the formation of a conditioning film (which forms within the first minutes due to organic molecules, i.e., proteins that stick to the surface). Then a primary film forms from a combination of slime of living and dead bacteria cells; further on microalgae, protozoa spores, and diatoms colonies increase the microfouling, forming a secondary film consolidated through secretion of natural adhesives. The latter stimulates the settlement of algae, animal larvae, and attachment of marine organisms, giving rise to the macrofouling. The evolution of the fouling depends on environmental conditions, changing with seasons, and daylight. The approaches to face the problem, known since ancient times, involve coatings with solubilization or not of active agents. The presence of soluble biocide species, such as tributyltin, extensively used from1950s, was revealed as quite dangerous for the marine environment, with local extinction of some species and bioaccumulation. Thus, strict environmental and health restrictions (issued by International Maritime Organization in 2001, with the International Convention on the Control of Harmful Antifouling Systems on Ships becoming effective in 2008) imposed a move toward alternative compounds, such as rosin (from coniferous trees), extracts of antagonist bacteria, and tin-free resins [95].

In the case of non-biocide release coatings, they can be divided into different categories: (a) coatings that detach biofoulants (i.e., hydrophobic materials, such as fluorocarbons, hydrocarbons, and polydimethylsiloxanes); (b) coatings that inhibit microbial growth [49,96]; (c) coatings that prevent attachment of biofoulants (such as PEG-based materials for their resistance to protein adsorption and cells adhesion, or antimicrobial peptides) [1,97,98,99]; or (d) materials able to create a superhydrophobic surface, based on ZnO NPs functionalized with hexadecyltrimethoxysilane [100], or pluronic-lysozyme conjugate coatings [101]. Thus, the measurement of microbial adhesive forces represents a crucial topic [102].

Coatings able to impart to steel long-term surface antifouling and anticorrosion properties have been prepared, one–pot synthesis of dedoped Br-polyaniline with epoxy resin (E51) [72], through the formation of an interpenetrating polymer network based on epoxy-acrylic-oleic acid with butylated melamine formaldehyde [103].

#### 2.3.2. Dental Sealers and Medicinal Applications

Nanotechnologies have been introduced to enhance oral health, including treatments of different problems, such as dentin hypersensitivity, biofilm removal, treatment of oral cancer, and endodontics to improve antimicrobial efficacy and tissue regeneration. In endodontic therapy, the elimination of microorganisms and prevention of re-contamination activity is accomplished through materials with suitable mechanical and chemical compatibility, and dentine adhesion long-lasting properties. Endodontic infection is polymicrobial with methicillin-resistant *Staphylococcus aureus* and vancomycin-resistant streptococci. Thus, introduction of organic nanoparticles (such as chitosan, eugenol, or poly-lactic-co-glycolic acid encapsulated with photoactive drugs) [45,104] or inorganic nanoparticles (such as bioactive calcium silicate, Au, Ag, ZnO, TiO_2_, MgO, or CaO NPs), have been tested in epoxy nanocomposite to prevent infections [104,105,106,107,108]. It was recently reported that ultrasonic activation of the AH plus sealer (based on diglycidyl ether of bisphenol-A, and 1-aminoadamantane together with N,N’-dibenzyl-5-oxanonandiamine-1,9 as coupling agents and containing ZrO_2_) improved the filling quality and significantly reduced the *Enterococcus faecalis* viability in superficial dentine [109].

One study looked at the effects of antibacterial activity of quaternary ammonium polyethylenimine nanoparticles (prepared through different protocols) incorporated at 2 wt% in different epoxy-amine root canal sealer pastes. It was observed that all of the materials exhibited good antibacterial and antibiofilm properties, which could be improved without compromising other physical properties, by tailoring the synthesis of the nanoparticles [81].

Conventional epoxy-based dental sealers have also been modified with bioactive glass, hydroxyapatite, and fluorine substituted hydroxyapatite to compare the antibacterial properties of the sealers. In all cases, a significant reduction of the biofilm formation was observed, even if the resin, added with fluorine substituted hydroxyapatite, was the most active against *Enterococcus faecalis,* and *Streptococcus mitis* [110].

Hinokitiol (Table 4), a natural derivative that displays strong antimicrobial activity, used in toothpaste and oral-care gels to limit the proliferation of microorganisms in the oral cavity, has recently been added to dental root sealers, either alone or in the presence of ZnO nanoparticles, resulting in dose-dependent inhibition of *Staphylococcus aureus* abscess formation [111].

#### 2.3.3. Fibers and Fabrics

As for coatings—antimicrobial activity has been studied not only for “flat” samples, but also for other materials, such as fabrics, and a synergistic effect between the chemical action of materials that constitute the coating and the surface topography, which influences the bacterial attachment, was observed [8].

Neem oil dissolved in epoxy resin and aged to nano-silica particles obtained by APTES, was cured in the presence of areca natural fibers, achieving epoxy-neem bio composites with improved antimicrobial behavior, even if viscoelastic and thermal stability was reduced. The composites have been proposed as containers for bio-food and medical drug storage [112].

The antimicrobial properties of livestock chicken feather fibers also improved, by reinforcement with cardanol benzoxazine-epoxy composites, achieving a bio-based environmentally friendly new material derived from renewable resources. It exhibited low dielectric behavior compared with conventional semiconductor insulator materials, and was light in weight with respect to conventional reinforcement composites [113].

Multifunctional fabric finishes have been achieved through electrospray processes using two different solutions: a N,N-dimethylacetamide/acetone solution of poly-vinylidene fluoride containing TiO_2_ NPs (P25, 21 nm) and N,N-dimethylacetamide/acetone solution of the epoxy resin (Epikote-828) and the curing agent triethylenetetramine. PET fabric was electrosprayed: the fibers resulted in being coated with microparticles with different deposition densities. The hydrophobic nature of poly-vinylidene fluoride, the presence of TiO_2_ NPs, and the cross-linked epoxy resin retained water droplets on the surface, without penetration into the fabric. The hydrophobic nature of the fiber surface (water contact angle of 152°) and the presence of TiO_2_ NPs produced antimicrobial activity, improved under UV light due to the activation of TiO_2_, suggesting that fabric finished with this treatment can be promising for the preparation of ultraviolet protection and self-cleaning materials [114].

Carbon fibers/epoxy composites with polyamide 6 (PA6) and 25% TiO_2_ were used to modify PA6 electrospun nanofiber veils. The energy absorbed during the crack propagation was improved and flexural strength increased by more than 24% with respect to the pristine PA6 fibers. Antibacterial properties, when irradiated with UV light (against *Escherichia coli* and other coliform bacteria) could be observed due to the photocatalytic effect of TiO_2_ [115].

Flax fiber yarn was modified by grafting ZrO_2_ NPs and then treating it with an epoxy resin based on diglycidyl ether of bisphenol F, cured with methylhexahydrophthalic anhydride, improving the mechanical properties of the fibers (i.e., tensile strength from 499.7 MPa of control fiber to 723.4 MPa for fibers grafted with sonication), and imparting antimicrobial properties tested against *Aspergillus niger* [116].

#### 2.3.4. Other Examples

The manufacturing of pressure-sensitive adhesives based on an epoxy resin obtained by the epoxidation of soya bean oil, reacting with acrylic acid and triethylamine under different experimental conditions, was studied in [74], where the authors reported antimicrobial activity against *Staphylococcus aureus*.

A new nanocomposite with unique antimicrobial and thermally conductive properties was prepared according to the following procedure: hexagonal boron nitride (h-BN) was bombarded by ion/electrons in a plasma forming OH@h-BN. Then, formic acid until pH 4 and 3-glycidoxypropyltrimethoxysilane (GYMO) was added. GYMO@h-BN-bearing epoxy groups reacted with trimethyl acetohydrazide ammonium chloride to give the final nanocomposite (AB@h-BN nanoplatelets), bearing different amounts of low melt alloys. A thermal conductivity increasing with the low metal alloys content, achieving 2.66 Wm^−1^K^−1^, accompanied by a significant antimicrobial activity against *Escherichia coli* and *Staphylococcus aureus* was observed, suggesting that these materials have great potential in the thermal management of medical electronic devices [117].

In a circular economy perspective, 1,4-benzene dicarboxamide derivatives, synthesized through depolymerization via aminolysis of polyethylene terephthalate waste, have been studied as curing agents in epoxy-resins formation, in the presence of isopropylamine or n-propylamine as catalyst and initiator, exhibiting potentially good antifungal activity [118].

## 3. Enhancement of Mechanical Properties via Nanomodification of Epoxies

In this section, we focus on the typical damage mechanisms occurring in polymer nanocomposites, followed by empirical evidence of the effect of nanomodification on the mechanical properties of epoxies. Nanofiller addition to polymers results in a remarkable increase in the initial fracture toughness, and a less pronounced increase of the elastic properties. Differently, the strength of nanomodified polymers strongly depends on the process and the considered material systems.

### 3.1. Damage Mechanisms within Polymer Nanocomposites

The mechanical properties of nanocomposites are undoubtedly related to the physical damage mechanisms taking place within them. Indeed, different reinforcement shapes, as well as different morphologies in the reinforcement distribution, are acknowledged to promote different damage mechanisms that, in turn, result in different mechanical properties (in particular strength, fracture toughness) [119].

Within this context, one important point to consider is related to the filler dispersion and distribution. Indeed, the filler “morphology”, i.e., clustered or homogenously dispersed and distributed, unavoidably affect the effective size of reinforcement, with direct consequences on the damage mechanisms.

Microscale toughening mechanisms (such as crack pinning, crack deflection, and microcracking, ahead of the main crack) derive from the mechanical interactions of the crack front with micrometer-sized reinforcements (such as micro-reinforcements and nanoreinforcement clusters).

Differently, nanoscale-toughening mechanisms take advantage of the nano-effect to bolster energy dissipation. In the case of spherical nanoparticles or nanoclay-filled polymers, the following damage mechanisms were observed to occur: nanoreinforcement debonding, matrix shear deformation, plastic yielding, and void growth. We should note that, in the same composite material, both nanoscale and microscale mechanisms can appear, either by using different types or reinforcements or by partial aggregation of nanoreinforcements.

Moreover, a grouping based on the nanoreinforcement shape is useful. There exist three main reinforcement shapes—nanoplatelets, nanotubes, and nanoparticles, characterized by having one, two, and three dimensions at nanometer scales, respectively. Depending on the shape, different nanoscale mechanisms may be dominant.

**Figure 1:**
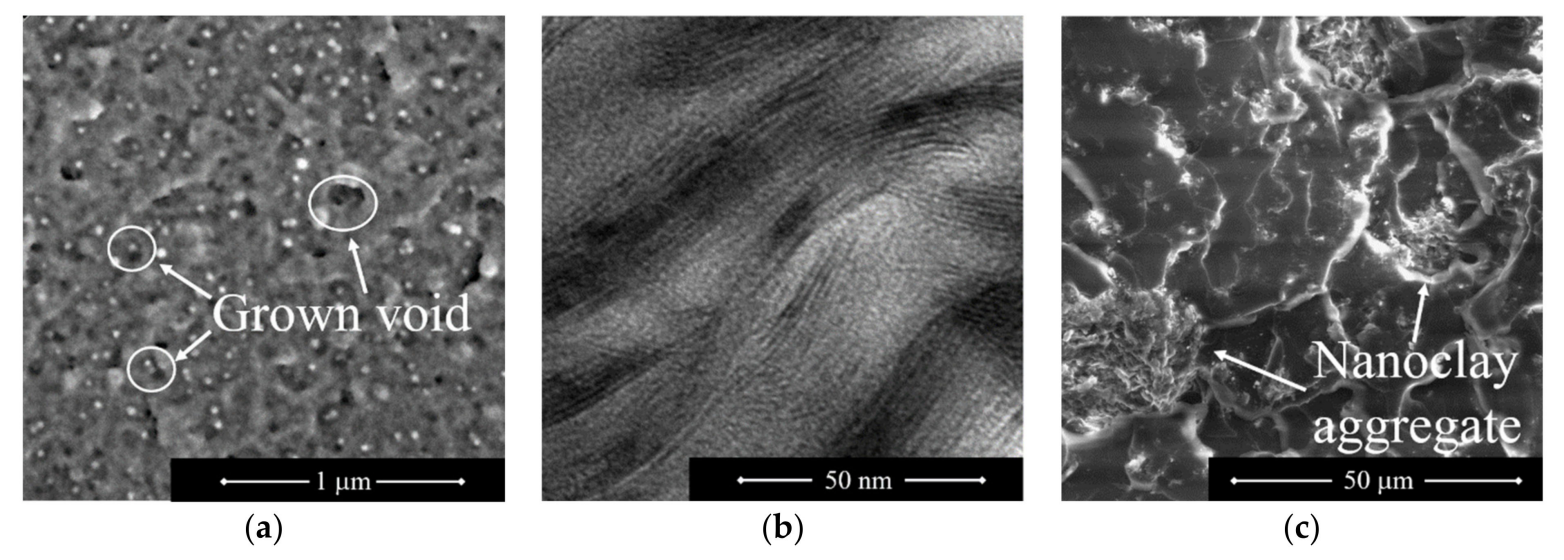
Micrographic pictures of nanoreinforced epoxies. (**a**) Fracture surface of a silica-nanoparticle reinforced epoxy. (**b**) TEM image from a montmorillonite reinforced epoxy. (**c**) Fracture surface of a nanoclay reinforced epoxy. These images were taken from the same batch of those used in [125,126].

Focusing on the mechanisms taking place at the nanoscale—the literature [120] reports a damage pattern in which, initially, debonding between some particles and the matrix takes place. This debonding is the catalyst for the other mechanisms, which are responsible for the main energy adsorption. In particular, some of the cavities resulting from debonding are expanded by plastic yielding of the polymer, whilst shear bands, due to the matrix shear yielding, may also appear. Grown voids are reported in Figure 1a in the case of spherical nanoparticles. Damage phenomena only affecting some particles can be justified by the statistical nature of damaging paired with the resulting local stress redistribution.

In the presence of nanoclays a different mechanism could take place, based on the characteristic morphology of this kind of reinforcement. Indeed, usually nanoclays are thin layers of clay stacked together to form so-called tactoids. One example of such a stacking is visible in Figure 1b, where a TEM image of a nanoclay-reinforced epoxy is depicted. While a single layer has a thickness in the order of 1 nm or lower, a tactoid may combine tens or hundreds of layers; hence, reaching significant thickness. Considering polymer-nanoclay interaction, clay layers may be isolated from each other, or the polymer may be able to infiltrate through tactoid layers without splitting them apart. In the first case, the morphology is named exfoliated, in the latter—intercalated. There is also a case where the polymer is not able to infiltrate through tactoid layers. Such a morphology is named segregated. While exfoliated morphologies promote the nanoscale damage mechanisms already presented, segregated morphologies promote microscale mechanisms. However, intercalated morphologies may promote a peculiar damaging scenario, where the crack front is able to move between two layers of a tactoid, splitting it apart [119]. In such a case, the interlayer mechanical properties (usually low) are combined with the deflection of the crack front, with an outcome that may result in nanocomposite toughening.

Concerning microscale damaging, crack deflection is a typical mechanism of the microscale, where the crack front is either tilted or twisted, increasing the total fracture surface, and resulting in mixed mode crack propagation, effectively increasing adsorbed energy [121]. Another mechanism at the microscale is crack pinning, where the crack front is unable to cut-through hard inclusions and is forced to propagate around them. This modification of the crack path results in a typical crack-front bowed shape, with the crack front splitting at the inclusion, and merging past it, forming a step in the crack path [122]. Another important mechanism is microcracking ahead of the main crack, where nanoreinforcement clusters act as stress concentrators, resulting in several micro-cracks ahead of the crack front [123]. One consequence of this phenomenon is stress redistribution within the crack process zone; hence, affecting the energy required by the crack growth. Figure 1c presents an example of a morphology with clustered nanoplatelets. As expected, the presence of microsized aggregates promoted microscale damage mechanisms.

One final, important concept to underline is that the presence of nanoreinforcement clusters is not, by itself, detrimental to the mechanical properties of the nanocomposite. If, on the one hand, clusters prevent nanoscale damage mechanisms to take place, then, on the other hand, aggregates may trigger microscale damage mechanisms, effectively compensating for the former point [124]. However, the identification of an optimal morphology for a nanocomposite is a challenging task, given the number of competing mechanisms and the presence of chemical interactions among its constituents.

### 3.2. Experimental Evidences of Mechanical Properties Enhancement

Over the last 20 years, several experimental studies were carried out to quantify the enhancements of polymer mechanical properties through nanomodification. The literature on this topic is so broad that a comprehensive literature review is far from easy to be carried out. Accordingly, without the ambition of being exhaustive, in the following, experimental evidence taken from the literature (and mostly related to previous works by the present authors) are reconsidered. These results are, however, in line with the findings of several other authors and, therefore, could be considered as representative examples.

**Figure 2:**
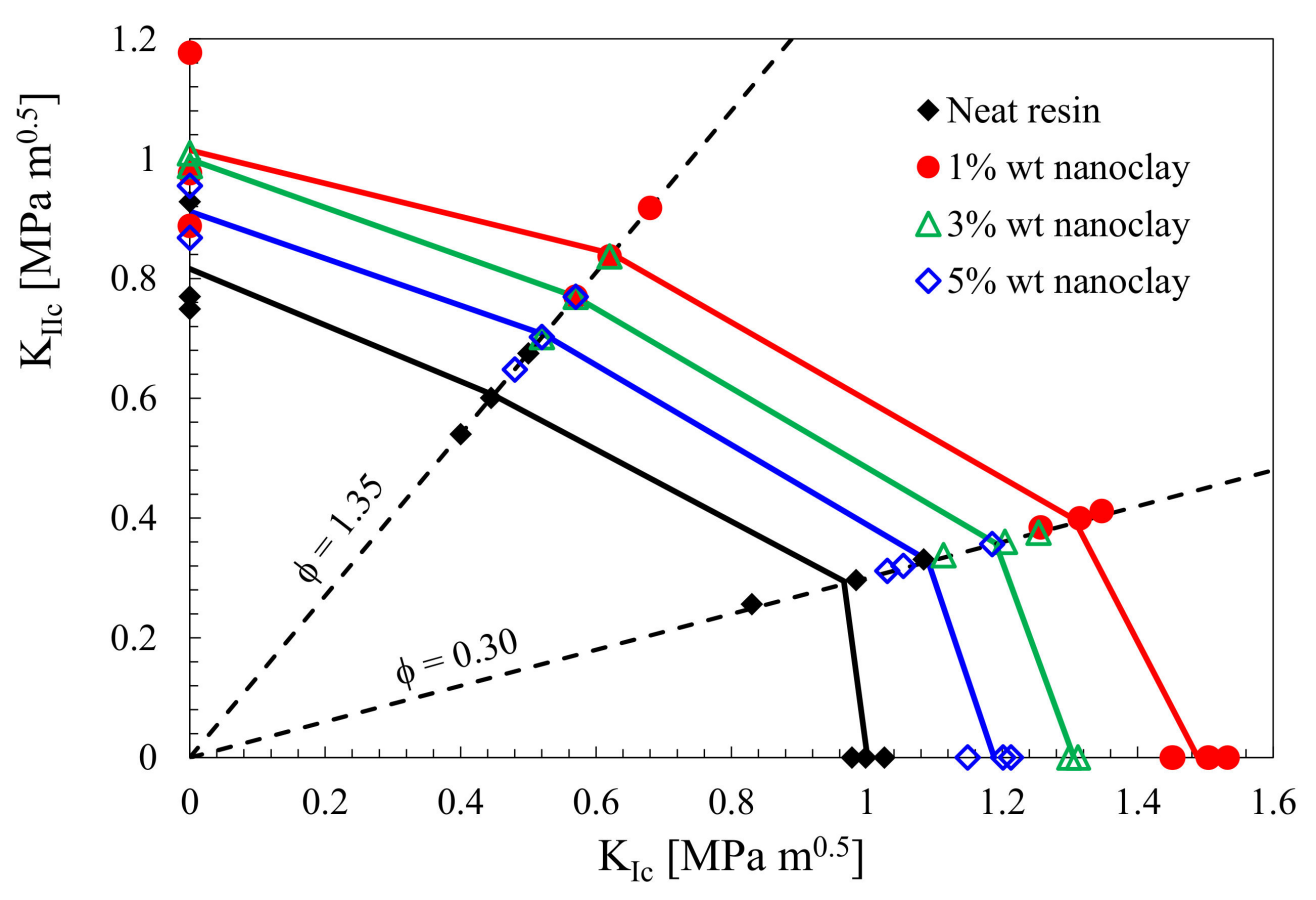
Fracture toughness of neat and nanomodified specimens under mode I, mode II, and mixed mode. Φ = K_II_/K_I_ is the mode mixity coefficient. DGEBA-based epoxy resin modified with montmorillonite nanoclay [127].

It is universally accepted in the literature that polymer nanomodification allows significant improvements in fracture toughness. Several papers report ameliorations from 30% to 60% of both mode I ([125,126,127]) and mode II [127] fracture toughness, with low nanofiller amounts, almost independently of the type of nanoreinforcement used. Usually fracture toughness increases with the filler content, up to a maximum value. Further addition of nanofillers cause levelling of the fracture toughness and, in some cases, even a steep decrease. For example in reference [127], the authors reported an increase of mode I fracture toughness, K_IC_, from about 1 MPa∙m^0.5^ in the case of a DGEBA unmodified epoxy up to about 1.5 MPa∙m^0.5^ with 1 wt% of montmorillonite nanoclay addition. For mode II fracture toughness—K_IIC_, from about 0.82 MPa∙m^0.5^ to about 1 MPa∙m^0.5^ for the same filler amount (see Figure 2). Similar results were observed in the case of mixed mode (I + II) fracture tests. From Figure 2, it is also evident that a further increase in nanofiller weight content resulted in a less pronounced increase of the fracture toughness, independent of the loading mode.

**Figure 3:**
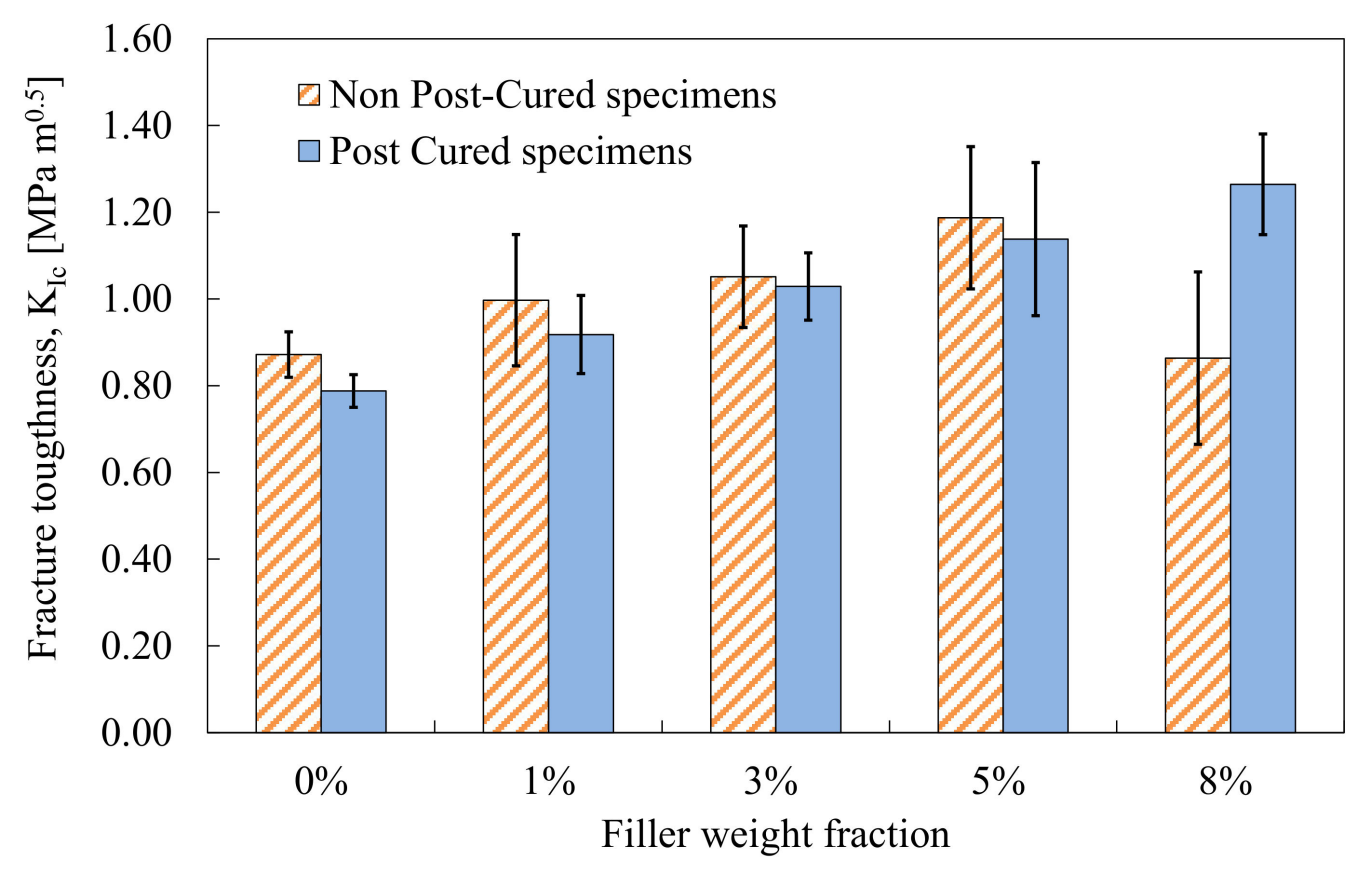
Mode I fracture toughness of neat and nanomodified specimens. DGEBA-based epoxy modified with silica nanoparticles. Post-curing was carried out in an oven at 60° C for 15 h [125].

From the analysis of the fracture surfaces, the authors noted very rough surfaces, with several micro-clusters and patterns associated with crack pinning and crack deflection. The results from mode I fracture tests on an epoxy resin modified with a masterbatch of silica nanoparticles are discussed in reference [125], where the importance of optimizing the curing cycle of the epoxy was highlighted. In particular, the authors showed that different curing cycles resulted in different fracture toughness values [125], obtaining a monotonic increase of K_IC_, with improvements up to +60% with 8 wt% of nanofiller (see Figure 3). Such a significant improvement was believed to be due to the homogeneous reinforcement distribution promoting diffuse toughening mechanisms, such as nanoparticle debonding and void growth.

**Figure 4:**
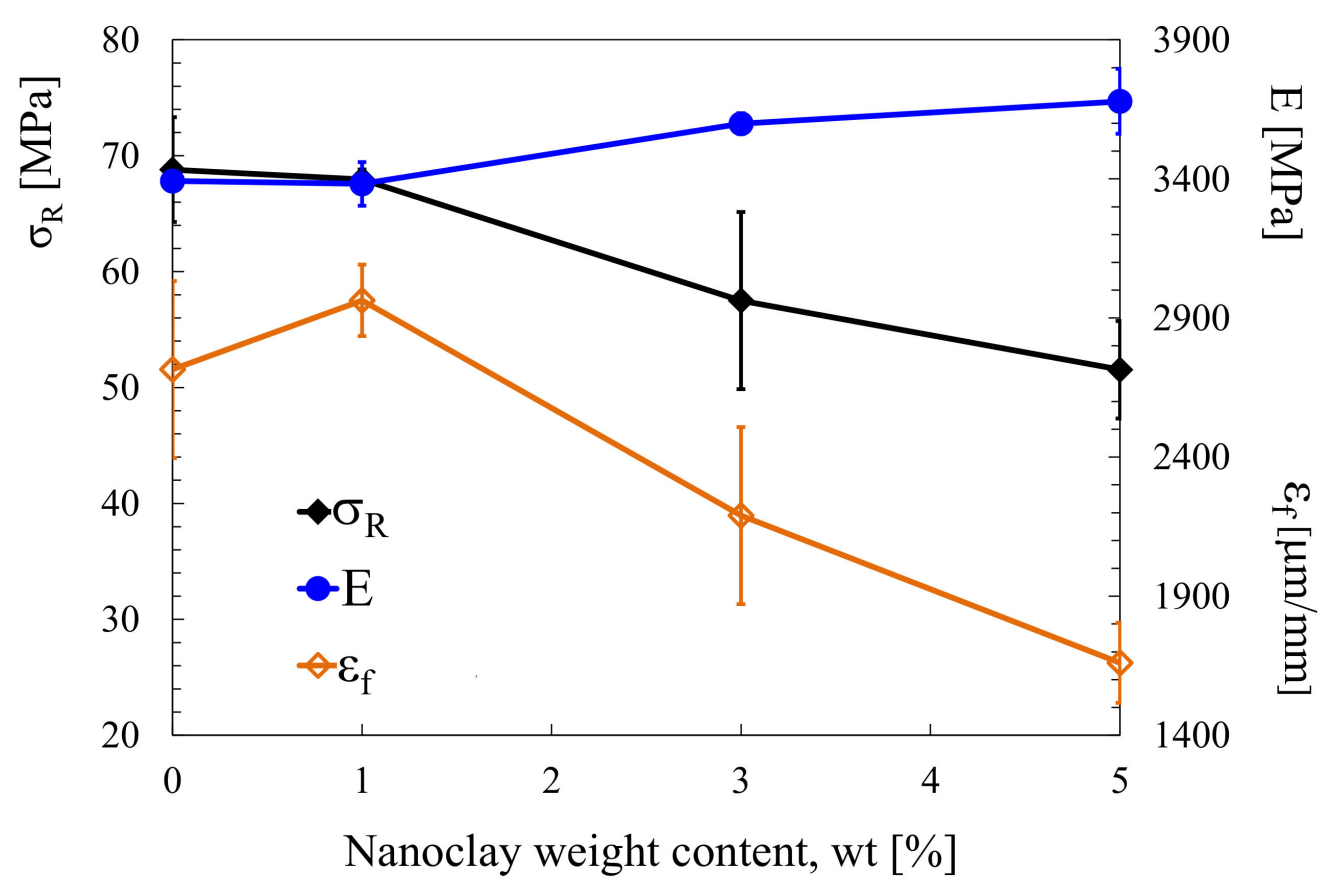
Tensile strength, strain to failure and Young modulus of neat and nanomodified epoxy resins. DGEBA-based epoxy resin modified with montmorillonite nanoclay [127].

Whilst the improvement of the fracture toughness appears to follow consistently nanoreinforcement addition, nanomodification does not guarantee a systematic increase of the polymer strength, even in the presence of satisfactory reinforcement distribution. For example, in reference [126,127,128], the epoxy resin strength was shown to decrease by nanoclay loading (see, as a representative example, Figure 4).

The same behavior was observed for epoxy/nanosilica nanocomposites in reference [125], where the authors minimized strength deterioration by means of a suitable curing cycle.

Regarding the elastic modulus—all of the papers previously mentioned reported negligible effects, usually with improvements from +0% up to +10% compared to neat-epoxy, proportional to the filler amount.

## 4. Enhancement of Mechanical and Antimicrobial Properties of Epoxy Composites

### 4.1. Epoxy Resins with Enhanced Mechanical and Antimicrobial Properties

The results reported so far highlight the possible benefits of adding nanoreinforcements to epoxy resins, in terms of antimicrobial or mechanical properties. The natural evolution of this research line is to combine both effects, developing multifunctional composites, displaying both antimicrobial and enhanced mechanical properties.

One attempt to create such synergy is reported in [126], where the authors used a DGEBA resin suitable for vacuum infusion of composite laminates as a matrix, and an octadecylamine montmorillonite nanoclay as a reinforcement. Eventually, they reported a systematic improvement in fracture toughness, K_IC_, proportional to nanoclay addition, reaching +30% at 3 wt% of nanoclay. At the same time, a slight increase in the elastic modulus and a more relevant decrease in tensile strength (−10%) were reported. One important result was the significant antimicrobial effect on the surfaces of samples manufactured with the modified epoxy, reaching a killing percentage of about 88% against *Escherichia coli* and *Staphylococcus aureus*.

A similar result is reported in [19], where the authors used a copper modified montmorillonite to modify the epoxy matrix. In this last mentioned case, fracture toughness, K_IC_, increased up to +25% with 1 wt% of nanoclay, while further addition resulted in a decrease of the enhancement (+15% at 3 wt%), in agreement with the results discussed in the previous section. Tensile strength steadily decreased when increasing the filler content (about −10% at 3 wt% of filler), whereas a slight reduction in the elastic modulus was noted. Regarding the antimicrobial properties—the addition of 3 wt% Cu^2+^-montmorillonite to the epoxy resulted in about 97% growth inhibition against *Escherichia coli* and close to 100% against *Staphylococcus aureus*.

In reference [10], the authors tested the effects of adding different natural Moroccan nanoclays on the mechanical and antimicrobial activity of an epoxy resin. The aim of the research was to study different clays naturally available in the Moroccan region for manufacturing multifunctional nanocomposites. Regardless of the type of nanoclay, fracture toughness, K_IC_, showed an increase in the range +10%/+40% with up to 5 wt% of nanoclay addition. Interestingly, the maximum value of K_IC_ amelioration depended not only on the nanofiller amount, but also on the specific nanofiller. Regarding the antimicrobial activity—the response of the obtained nanocomposites strictly depended on the system used, ranging from a limited effect against *Escherichia coli* and *Staphylococcus aureus*, to a significant killing rate (close to 100%).

### 4.2. Fiber Reinforced Laminates with Enhanced Mechanical and Antimicrobial Properties

The possibility of significantly improving the antibacterial properties of composite laminates, without compromising their mechanical performances, was proven in reference [126], where Double Cantilever Beam (DCB) and Inter-Laminar Shear Strength (ILSS) tests were performed on glass fiber reinforced laminates manufactured with neat and nanomodified epoxy matrix. In particular, it was shown that, for the studied system, nanomodification had a negligible effect on the interlaminar fracture toughness, G_IC_, of the laminates, and a limited detrimental effect on ILSS. However, the laminates were characterized by a significant antimicrobial effect with a maximum killing percentage of 94% against *Staphylococcus aureus* and 97% against *Escherichia coli* in the case of 3 wt% of nanofiller content.

**Figure 5:**
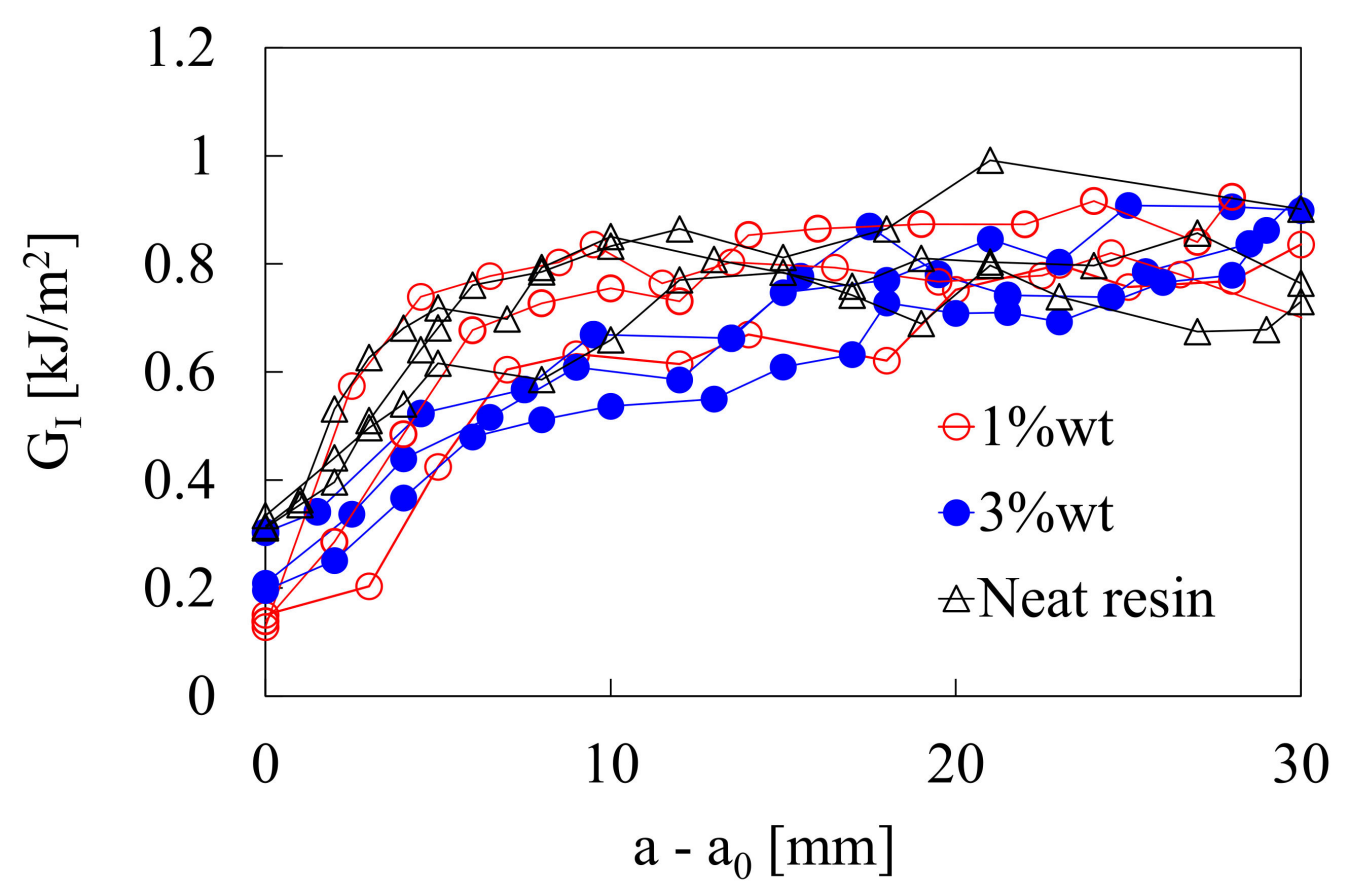
R-curves obtained from DCB specimens made of neat and nanomodified laminates [19].

In reference [19], nanomodified glass fiber laminates were manufactured with 1 wt% and 3 wt% of Cu^2+^-montmorillonite and their mechanical and biocide properties were thoroughly investigated. Representative examples of the results of DCB tests are shown in Figure 5, where it is evident that nanomodification has a very limited detrimental effect on the initiation value of the interlaminar toughness, whereas no significant effect could be noted on the propagation value.

Regarding the antibacterial properties, laminates manufactured with 3 wt% of nanofiller content exhibited very pronounced antibacterial activity. In particular, a 97% growth inhibition against *Escherichia coli* and a value close to 100% against *Staphylococcus aureus* were measured. This means that the transfer of antibacterial properties from the nanomodified epoxy to the laminates was successful.

## 5. Concluding Remarks and Future Research Trends

In this work, a comprehensive overview of the possibility of obtaining, through nanomodification, multifunctional epoxies with improved antimicrobial activity and enhanced mechanical properties, was presented.

To this end we focused on the most studied approaches to achieve epoxy nanocomposites exhibiting antimicrobial activity, including methods used to evaluate their efficacy against bacteria and fungi, which, even if based on standard parameters, must be often optimized with respect to the specific material and microorganism. In addition, relevant examples of industrial applications were provided, such as antifouling and anticorrosion coatings for hulls, dental sealers and connected applications, fibers and fabrics, and adhesives.

We then focused on mechanical properties of this class of multifunctional materials, presenting a discussion on the typical damage mechanisms occurring in polymer nanocomposites, and providing quantitative examples of the possible ameliorations that could be obtained on their mechanical properties.

The possibility of exploiting nanomodification to improve both antimicrobial and mechanical properties of epoxies was analyzed and discussed through examples taken from the literature, relating to nanomodified polymers (binary nanocomposites) and nanomodified fiber-reinforced plastics (ternary nanocomposites).

We should note that, in the authors’ opinions, a detailed study would be necessary to understand the mechanisms of cell death on antimicrobial surfaces, including cellular communication mechanisms, to help identify new disinfection strategies and design new antibiotics with improved activity against pathogens. Nevertheless, it is practically impossible to compare, quantitatively, the antimicrobial activities of the different materials based on the literature, even if it is boundless, owing to the variety of specimens, testing procedure, and methods to achieve quantification. Nonetheless, the worrying reports published on resistance against silver nanoparticles [44] and ZnO nanorods [129], due to genomic changes in microorganisms [130], the increasingly widespread onset of contact dermatitis (with epoxy resins, too) [131], and the fact that bisphenol A could be absorbed via dermal contact (from epoxy resin lining for food and beverage containers and thermal papers) and enter in the metabolic system within 48 h, with possible oxidative DNA damage to humans [132], increases the need for academic research engagement.

## Figures and Tables

**Table 1:** Schematic description of selected standard methods for antimicrobial susceptibility of surfaces.

Standard Method, Title	Test Organisms		Notes
	Bacteria	Fungi	
**Agar Diffusion Methods**			
AATCC Test Method 147-2004 Antibacterial Activity Assessment of Textile Materials: Parallel Streak Method	*Staphylococcus aureus,* *Klebsiella pneumoniae*		Measurement of inhibition zone after incubation in agar culture in the presence of the test specimen. Designed for diffusible antimicrobial agents.
AATCC Test Method 30-2004 Antifungal Activity, Assessment on textile Materials: Mildew and Rot Resistance of Textile Materials		*Aspergillus niger*; *Penicillium varians*; *Trichoderma viride*	Evaluation using a microscope (50×) of the extent of fungal growth on the test specimens (discs).
BS EN 1104-2018 Paper and board intended to come into contact with foodstuffs-Determination of the transfer of antimicrobial constituents	*Bacillus subtilis*	*Aspergillus niger*	Evaluation of the diameter of inhibition zone around each specimen disc.
BS EN ISO 20645/AATCC 90 Textile fabrics, determination of antibacterial activity—Agar diffusion test plate	*Staphylococcus aureus,* *Escherichia coli* *Klebsiella pneumoniae*		Measurement of the inhibition zone after incubation in agar culture in the presence of the test specimen (< or >1 mm).
BS ISO 16869 Plastics assessment of the effectiveness of fungistatic compounds in plastic formulations		*Aspergillus niger* *Chaetomium globosum* *Penicillium funiculosum* *Trichoderma longibrachiatum*	Observation of fungal growth after pouring the inoculated molten nutrient–salt agar onto the surface of the base agar and test specimen to form a thin second layer.
ASTM E2722-14 Standard test method for using seeded-agar for the screening assessment of antimicrobial activity in fabric and air filter media	*Staphylococcus aureus* *Serratia marcescens*	*Aspergillus brasiliensis*	Designed to qualitatively evaluate the presence of antibacterial and antifungal activity in (or on) fabrics, or air filter media, simulating actual use conditions.
E2922-15 Standard guide for the use of standard test methods and practices for evaluating antibacterial activity on textiles	*Staphylococcus aureus,* *Escherichia coli* *Klebsiella pneumoniae*	*Candida albicans*	An index of procedures.
**Suspension Methods**			
E2149-20 Standard methods for determining the antimicrobial activity of antimicrobial agents under dynamic contact conditions (fabric, paper, powder and granular material, surfaces)	*Escherichia coli*		Enumeration of CFU after contact between pieces of specimen with known bacterial concentration on the wrist-action shaker. Designed for non-diffusible antimicrobial agents.
AATCC test method 100-2004 antibacterial finishes on textile materials: assessment of	*Staphylococcus aureus,* *Klebsiella pneumoniae*		Designed to measure the antimicrobial activity of textiles after direct inoculation of the textile surface.
JIS L 1902 Testing antibacterial activity and efficacy on textile products	*Staphylococcus aureus, Klebsiella pneumoniae*		Final microbial concentrations are determined after test fabrics are allowed to incubate undisturbed in sealed containers at body-temperature for 18 h.
ISO 20473-2013 Textile determination of antibacterial activity of textile products	*Staphylococcus aureus, Klebsiella pneumoniae*		Three inoculation methods are proposed: (a) Absorption method (an evaluation method in which the test bacterial suspension is inoculated directly onto specimens); (b) Transfer method (test bacteria are placed on an agar plate and transferred onto specimens); (c) Printing method (test bacteria are placed on a filter and printed onto specimens).
**Quantitative Methods Based on Direct Contact of Microorganisms with Surfaces**			
JIS Z 2801-2000 Antimicrobial product test for antimicrobial activity and efficacy for plastics	*Escherichia coli* *Staphylococcus aureus,*		Enumeration of CFU (after serial dilutions) in the test inoculum applied to the test surface.
ISO 22196/JIS Z 2801 Plastic measurement of antibacterial activity on plastic surfaces	*Escherichia coli* *Staphylococcus aureus*		Enumeration of CFU (after serial dilutions) in the test inoculum applied to the test surface.
ASTM E2180-18 Standard test method for determining the activity of incorporated antimicrobial agent(s) in polymeric or hydrophobic materials	*Staphylococcus aureus* *Klebsiella pneumoniae, Pseudomonas aeruginosa*		Designed to measure the antimicrobial activity of highly hydrophobic surfaces. The vehicle for the inoculum is an agar slurry, which reduces the surface tension of the saline inoculum carrier and allows formation of a “pseudo-biofilm”, providing more uniform contact of the inoculum with the test surface. The method can confirm the presence of antimicrobial activity in plastics or hydrophobic surfaces and allows determination of quantitative differences in antimicrobial activity between untreated plastics or polymers and those with bound or incorporated low water-soluble antimicrobial agents.
**Methods Based on the Biofilm Formation**			
E3151-18 Standard test method for determining antimicrobial activity and biofilm resistance properties of tube, yarn or fiber specimens	*Staphylococcus epidermidis*		Determination of planktonic bacteria and adherent bacteria populations that survived exposure to test specimens. Designed for plastic surfaces and other non-porous surfaces including coated substrates

**Table 2:** Selected inorganic antimicrobial metal-based nano-systems in epoxy resins.

Material	Size and/or Morphology	Test Microorganism	Application	Reference
Ag NPs	100 nm	*Escherichia coli, Staphylococcus aureus, Bacillus subtilis, Salmonella typhi, Candida albicans,* *Aspergillus niger*	Hygienic pharmaceutical packaging	[35]
β-Ca_3_(PO_4_)_2_	20–550 nm	*Escherichia coli,* *Enterococcus faecalis*	Dental sealer	[45]
Ag/TiO_2_	20–100 nm	*Escherichia coli,* *Staphylococcus aureus*	Bactericidal surfaces	[46]
Ag/APTES	>15 nm	*Pseudomonas aeruginosa,* *Bacillus subtilis, Escherichia coli,* *Candida albicans*	Anticorrosive and antifouling coating	[44]
Ag/TiO_2_	20–100 nm	*Streptococcus mutans*	Dental sealer	[47]
AgO	100 nm, quadrangle	*Escherichia coli,* *Staphylococcus aureus*	Steel protection in the marine environment	[48]
TiO_2_	<100nm	*Escherichia coli*, *Staphylococcus aureus*, *Cyclotella* sp.	Antifouling coating	[49]
Ag/TiO_2_	Flower-like		Self-cleaning, photocatalytic, antimicrobial coatings in public areas	[50]
Fe_3_O_4_/Fe_2_O_3_	<20 nm	*Escherichia coli, Staphylococcus aureus, Bacillus subtilis,* *Pseudomonas aeruginosa*	Coating on carbon steel	[51]
Ag	<20 nm	*Escherichia coli*	Nanocomposites with improved mechanical properties	[52]
ZnO	<20 nm	*Enterobacteriaceae*	Food packaging	[53]
CuO/TiO_2_	<25 nm	*Escherichia coli*	Corrosion and antibacterial protection coatings	[54]
Ag	<20 nm	*Listeria monocytogenes*	Nanocomposites with improved antimicrobial properties	[55]

**Table 3:** Some compounds in plant extracts used as antimicrobial additives in epoxy resins.

Compound	Description
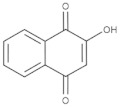	Lawsone In the leaves of the henna plant (*Lawsonia inermis*), and in the flower of water hyacinth (*Eichhornia crassipes*).
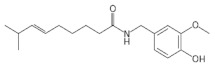	Capsaicin An active component of chili peppers.
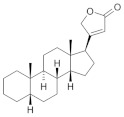	Cardenolide Many plants contain cardenolide derivatives and are used by some plant and animal species as defense mechanisms.
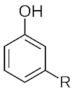	Cardanol From anacardic acid, it is the main component of cashew nutshell liquid.
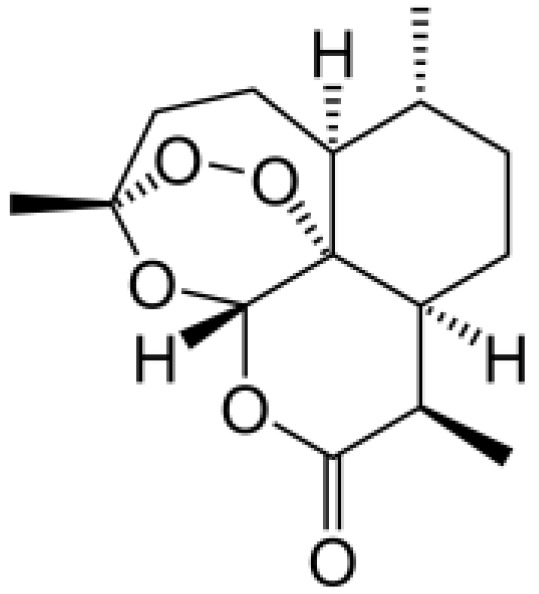	Artemisinin Extracted from the plant *Artemisia annua*, sweet wormwood, an herb employed in Chinese traditional medicine.

**Table 4:** Hinokitiol, a compound used as antimicrobial additive in medicinal applications.

Compound	Description
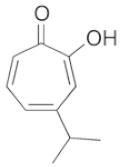	Hinokitiol A natural monoterpenoid present in the wood of trees in the family of Cupressaceae.

## Data Availability

No new data were created or analyzed in this study. Data sharing is not applicable to this article.
